# Urodynamic outcomes after pelvic nerve-sparing radical hysterectomy with or without neoadjuvant chemotherapy

**DOI:** 10.18632/oncotarget.27147

**Published:** 2019-08-27

**Authors:** Satoshi Tsunetoh, Yoshito Terai, Masaaki Takai, Satoe Fujiwara, Yoshimichi Tanaka, Tomohito Tanaka, Hiroshi Sasaki, Naokazu Ibuki, Takanobu Ubai, Kazuhiro Yamamoto, Haruhito Azuma, Masahide Ohmichi

**Affiliations:** ^1^ Department of Obstetrics and Gynecology, Osaka Medical College, Takatsuki, Japan; ^2^ Department of Urology, Osaka Medical College, Takatsuki, Japan; ^3^ Department of Radiology, Osaka Medical College, Takatsuki, Japan; ^4^ Department of Obstetrics and Gynecology, Kobe University, Kobe, Japan; ^5^ Department of Urology, Takatsuki General Hospital, Takatsuki, Japan

**Keywords:** uterine cervical cancer, nerve-sparing radical hysterectomy (NSRH), urodynamic study, neoadjuvant chemotherapy, balloon-occluded arterial infusion (BOAI)

## Abstract

**OBJECTIVE:** Our purposes of this study were to characterize a group of bulky cervical cancer patients who underwent a nerve sparing radical hysterectomy (NSRH) with or without neoadjuvant chemotherapy (NAC), to compare surgical outcomes and the preservation of bladder function, and to compare prognoses.

**RESULTS:** Fifty-three patients had NSRH without NAC (Group A), and 33 patients had NSRH after NAC (Group B). With regard to prognostic factors, there was only a significant difference between both groups with regard to lymph node metastasis (15% vs 42%, *P* = 0.01). Moreover, bladder function in Group B patients improved to the same extent as the preoperative rate three months postoperatively. These data were similar to the results in Group A. With regard to overall survival, the 5-year survival rate was 98.1% (95% confidence interval (CI) 87.8–99.7) in Group A and 86.7% (95% CI 71.7–96.7) in Group B (*P* > 0.1).

**METHODS:** We retrospectively identified 86 patients with cervical cancer who underwent NSRH at Osaka Medical College from May 2009 to November 2016. NAC was performed via balloon occluded arterial infusion. We extracted data on the patient's stage of progress, tumor volume, histological subtype, bleeding volume, urodynamic study results, and postoperative complications. The data were divided into two groups - those patients who received NAC and those who did not - and then compared.

**CONCLUSIONS:** According to our analysis, NSRH surgery after NAC via balloon occluded arterial infusion brings beneficial results to patients with bulky IB2 to IIB cervical cancers.

## INTRODUCTION

It is well known that, in cervical cancer patients, the function of the urinary tract can be affected by radical hysterectomy [1–5]. The most frequent complication is bladder dysfunction. The quality of life of patients who have undergone a radical hysterectomy is grown worse by the physical and mental stress caused by difficulty in urination. The reported incidence of impaired bladder function at 12 months after a radical hysterectomy is as high as 63% for sensory loss, 55% for stress incontinence, 85% for urination with abdominal pressure, as well as 63% for abnormal compliance [6]. Various attempts for preserving urinary function have been made, and investigators have recently reported various autonomic nerve preserving radical hysterectomy techniques. [7–11]. However, it has been criticized that there is no standardized technique for nerve sparing radical hysterectomy (NSRH), and controversies still exist as to its oncological safety [12–14]. Although there are reports that have included stage IIB cancers, they generally cover stage IB and IIA [15]. To date, however, there have been no reports on urinary function in which urodynamics are compared between NSRH and NSRH after neoadjuvant chemotherapy (NAC) for bulky cervical cancer. In this study, we compared the urodynamic results of radical hysterectomy with autonomic nerve preservation in cervical cancer patients with or without NAC.

## RESULTS

A total of 128 patients who underwent a radical hysterectomy during the period from May 2009 to Nov 2016, and in whom urodynamic examinations were conducted before the radical hysterectomy preoperatively and at 1 week and 1, 3, and 6 months postoperatively, were included in this study. We excluded 42 patients who did not undergo any urodynamic study at any period.

We compared the outcome of NSRH with or without neoadjuvant chemotherapy. In the study, 53 patients had NSRH without balloon occluded arterial infusion (BOAI) (Group A). (The details of which are described in the Materials and Methods section). Thirty-three patients had NSRH with BOAI (Group B). The mean age was 46.2 years (range, 31–70 years) for Group A and 50.8 years (range, 27–69) for Group B. Patient and tumor characteristics are presented in Table 1. In Group A, there were three patients with stage IA2 cancer, 44 patients with stage IB1, two patients with stage IB2, and four patients with stage IIA1 uterine cervical cancer. In Group B, there were four patients with stage IB2 cancer, six patients with IIA2, and 23 patients with stage IIB uterine cervical cancer. There were significant differences between each stage. Before receiving neoadjuvant chemotherapy, we checked tumor volume (major axis tumor volume) by magnetic resonance imaging. The mean tumor volume in Group A and Group B was 18.4 mm and 48.1 mm, respectively (*p* < 0.001), and thus showing a significant difference between both groups. However, after receiving NAC, we measured the tumor with excised specimen volume. The mean size of Group B decreased from 48.1 mm to 13.6 mm (*P* = 0.006), and the mean size of tumor was 20.3 mm in Group A. Apart from the length of surgery (median duration, 470 vs 461 minutes, *P* = 0.49), there were no significant differences between the two groups. Moreover, there was no significant difference in severe blood loss between the two groups (389 ml vs 400 ml *P* = 0.98), and other intraoperative complications (urinary tract injury, bowel injury, deep vein thrombosis, etc.) did not occur. There were also no postoperative complications in both groups. With respect to the known prognostic factors (histological subtype, invasion depth, presence of lymph-vascular space invasion, and linear extension), there were no significant differences between the two groups. There was a significant difference, however, between both groups with regard to lymph node metastasis (15% vs 42%, *P* = 0.01).

**Table 1:** Characteristics of patients of cervical cancer who underwent nerve-sparing radical hysterectomy with or without neoadjuvant chemotherapy

	*n* = 53	*n* = 33	
	without BOAI	with BOAI	
**age, mean (range), y**	46.2 (31–70)	50.8(27–69)	**0.06**
**Histological subtype, *n* (%)**			
**Squamous cell**	29(54.7)	25(75.8)	**0.046**
**adeno**	17(32.1)	6(18.2)	**0.15**
**adenosquamous**	3(5.7)	1(3)	**0.56**
**other**	4(7.5)	1(3)	**0.38**
**before NAC Stage, *n* (%) FIGO stage**			
**IA2**	3(5.7)	0(0)	
**IB1**	44(83.0)	0(0)	
**IB2**	2(3.8)	4(12.1)	
**IIA1**	4(7.5)	0(0)	
**IIA2**	0(0)	6(18.2)	
**IIB**	0(0)	23(69.7)	**<0.001**
**tumor volume (before therapy)**			
**MRI (Major axis) mean range**	18.4(0–41)	48.1(30-66)	**<0.001**
**tumor volume (after therapy)**			
**Maximum Linear extension, mean [SD], mm**	20.3[1.56]	13.6[10.8]	**0.006**
**Blood loss, median, (range), mL**	389(90–1850)	400(70–1720)	**0.98**
**Length of surgery, median, (range) min**	470(375–695)	461(313–570)	**0.48**
**Adjuvant therapy**			
**Chemotherapy, *n* (%)**	24(45.3)	17(51.5)	**0.57**
**Radiotherapy, *n* (%)**	9(17.0)	7(21.2)	**0.62**
**None, *n* (%)**	20(37.7)	9(27.3)	**0.31**
**Perioperative complications**	0	0	
**Postoperative complications**	0	0	
**Lymph space and vascular invasion, *n* (%)**	13(24.5)	4(12.1)	**0.18**
**No. removed nodes, mean (range)**	40(18–64)	37.2(15–79)	**0.15**
**The number of lymph node metastasis**	8(15)	14(42)	**0.01**
**Local recurrence, *n* (%)**	1 (1.9)	1 (3.0)	
**Total recurrence, *n* (%)**	1 (1.9)	4 (12.1)	**<0.05**
**Time to recurrence, mean [SD], mo**	5	6.5[2.6]	
**Follow-up, mean (range) [SD] mo**	96.2[24.2]	69.3[26.4]	**0.08**

**Figure 1:**
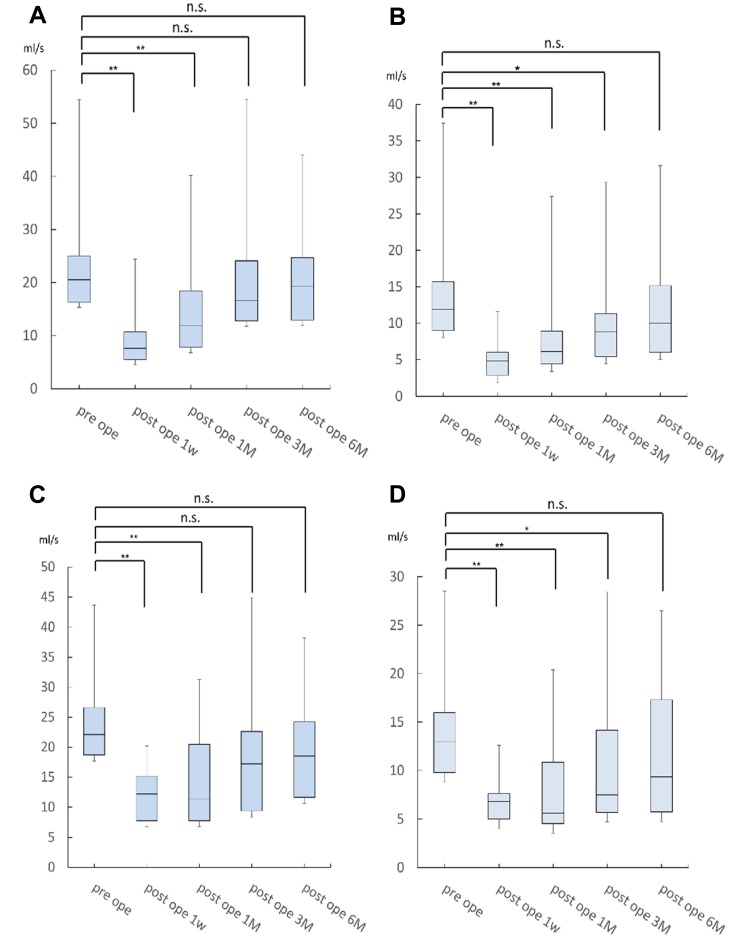
The result of Qmax rates for NSRH without NAC. There were significant differences between the preoperative Qmax rate and the 1 week and 1 month postoperative rates. However, there was no significant difference between the preoperative Qmax rate and the 3-month postoperative rate (*P* = 0.44) (**A**). These results indicate that the Qave of patients at 6 months improved to the same extent as the preoperative rate (*P* = 0.47) (**B**). Similarly, the results of the Qmax and Qave studies in patients who received NAC were equivalent: the Qmax of patients at 3 months improved to the same extent as the preoperative rate (*P* = 0.17) (**C**). The Qave of patients at 6 months improved to the same extent as the preoperative rate (*P* = 0.47) (**D**).

**Figure 2:**
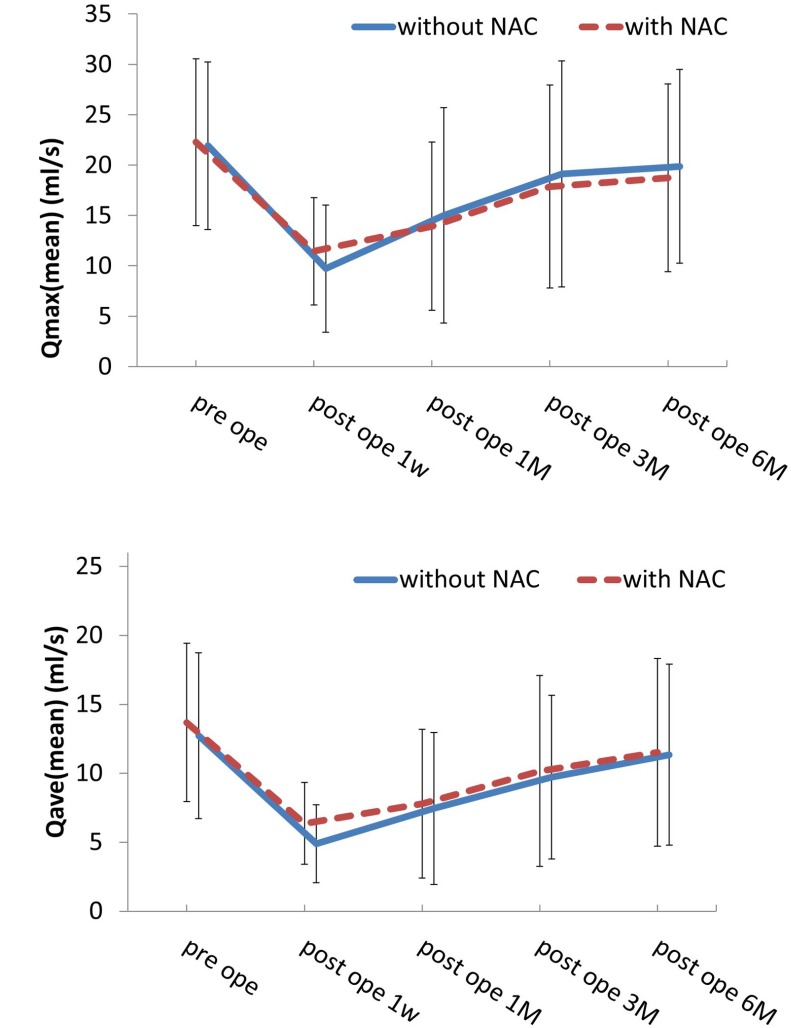
These results show that the progress of both Qmax and Qave was almost similar and that there was no significant difference between the two rates.

The results of the urodynamic study are shown in Figure 1. One month after surgery, if the residual urine volume was over 100 ml, the administration of drugs for urinary dysfunction, distigmine bromide (Ubretid^®^) and urapidil (Ebrantil^®^), was started. The medication was discontinued when the residual urine volume was less than 100 ml. The number of patients who used the medication for urinary dysfunction one month postoperatively was 22/53 (41.5%) for Group A and 11/33 (31.4%) for Group B. Three months after the operation, Group A was 10/53 (18.9%) and Group B was 3/33 (9%). There were no significant differences between the two groups. Figure 1A shows the results of the Qmax urinary flow rate in NSRH without NAC. There were significant differences between the preoperative Qmax and the rate 1 week and 1 month after operation; however, there were no significant differences between the preoperative Qmax and the 3-month (*P* = 0.44) and 6-month (*P* = 0.68) postoperative rates (*P* = 0.44). These Qmax results indicate that the Qmax of patients at 3 months improved to the same extent as the preoperative rate. Similarly, the results from the Qave study indicate that the Qave of patients at 6 months also improved to the same extent as the preoperative rate (*P* = 0.47) (Figure 1B). Similarly, as well, the results of the Qmax and Qave studies with patients who underwent NAC were equivalent; the Qmax of patients at 3 months improved to the same extent as the preoperative rate (*P* = 0.17) (Figure 1C). Moreover, the Qave of patients at 6 months also improved to the same extent as the preoperative rate (*P* = 0.47) (Figure 1D). We compared the progress of urodynamic results in Group A and B, and Figure 2 shows that the progress of both Qmax and Qave was almost similar and that there were no significant differences between the two groups.

**Figure 3:**
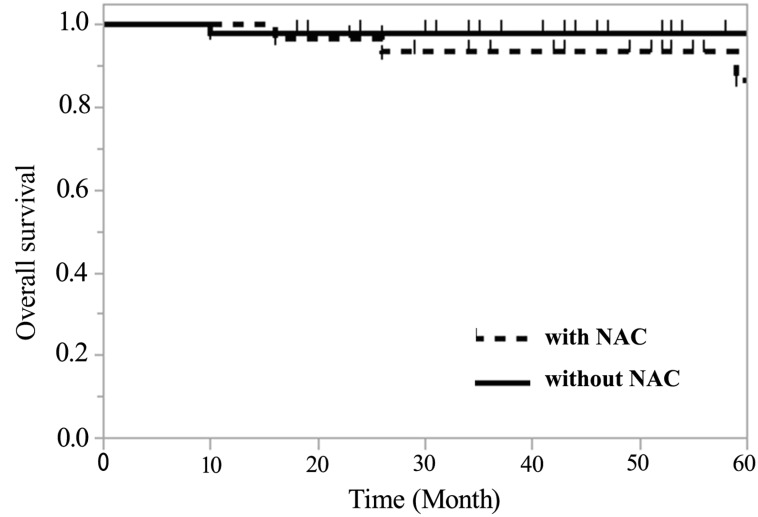
Regarding overall survival, the 5-year survival rate of Group A was 98.1% (95% confidence interval (CI) 87.8–99.7) and 86.7% (95% CI 71.7–96.7) in Group B, thus showing no significant difference.

The recurrence rate in Group A was 1.9% and 12.1% in Group B, thus indicating that there was a significant difference between both groups (*P* < 0.05). However, with regard to overall survival, the 5-year survival rate was 98.1% (95% confidence interval (CI) 87.8–99.7) in Group A and 86.7% (95% CI 71.7–96.7) in Group B (Figure 3), thus showing no significant difference.

## DISCUSSION

This study postoperatively investigated urinary function outcomes after NSRH with or without NAC in patients with cervical cancer. The results showed that urinary function, as measured by Qmax and Qave, recovered at 3 months and 6 months after NSRH without NAC. In bulky cervical cancer, after NSRH with NAC, urinary function showed equivalent results.

Patients with bulky stage IB2 to stage IIB cervical cancers have large tumor masses. There have been several literatures about intravenous NAC for advance cervical cancer. In Phase II studies, many other literatures have reported using cisplatin-based neoadjuvant chemotherapy in cervical carcinoma (Table 2). Bolis *et al*. reported on 79 patients with IB2-IIB tumors using cisplatin and ifosfamide [16]. Lai *et al*. reported on 59 patients with IB-IIB tumors treated with cisplatin, vincristine and bleomycin [17]. Choi *et al*. also reported on 46 patients with IB2-IIB tumors using cisplatin, mitomycin and vincristine [18]. Shoji *et al*. reported on 15 patients with IB2-IIB tumors using cisplatin and irinotecan [19], and Matunnura *et al*. reported on 46 patients with IB2-IIB tumors also using cisplatin and irinotecan [20]. The response rates in these studies were from 70.0 to 86.7% [16–20].

**Table 2:** Summary of neoadjuvant chemotherapy with intravenous for cervical cancer

Author	Year	N.P.	FIGO Stage	Regimen	R.R.(%)
Bolis, *et al.* [20]	1996	79	IB2-IIB	CDDP+IFM	70
Lai, *et al.* [21]	1997	59	IB-IIA (bulky)	CDDP+VCR+BLM	81
Choi, *et al.* [22]	2007	46	IB2-IIB	CDDP+MMC+VCR	83
Shoji T, *et al.* [23]	2010	15	IB2-IIB	CDDP+CPT-11	87
Matumura, *et al.* [24]	2010	46	IB2-IIB	CDDP(or NDP)+CPT-11	80

N.P.; Number of patients R.R.; Response rate.

CDDP; cisplatin IFM; ifosfamide VCR; vincristine BLM; bleomycin MMC; mitomycin C.

CPT-11; irinotecan NDP; nedaplatin.

There have also been several reports about intra-arterial neoadjuvant chemotherapy. The procedure of transarterial chemotherapy via transarterial chemoembolization (TACE) has been offered as a main treatment for liver or other cancers, and Tsubamoto H *et al*. showed the advantage of neoadjuvant TACE in cervical cancer [21]. Our previous study [22] reported on 60 patients with IIB-IVA tumors using cisplatin and mitomycin-C with pirarubicin hydrochloride via BOAI. The response rate of BOAI in this study was 96.7%. Another one of our studies [23] reported on 94 patents with IB2, IIA2, and IIB tumors using cisplatin and irinotecan via BOAI. The response rate of BOAI in this study was 92.6%. Patients who received BOAI and who had no response to the treatment received CCRT after NAC. Recently, Ujihara *et al*. reported on 52 patients with IB2-IIB tumors using intra-arterial neoadjuvant chemotherapy, and the overall positive response rate was 88.5% [24]. A retrospective analysis was performed on 93 cases of intravenous and 118 cases of intra-arterial neoadjuvant chemotherapy for stage IB2-IIB cervical carcinomas. The response rate for intravenous was 84.9% and 88.2% for intra-arterial administration [25]. These reports suggest that intra-arterial neoadjuvant chemotherapy is better than intravenous chemotherapy with regard to response rates. In our study, the overall positive response rate was 92.6%, and the tumors’ volume was reduced by an average of 71.7%. In this study, there are biases between Group A and B with regard to tumor size. The mean tumor size of Group A was 18.4 mm, and this group consisted mainly of stage IB1 patients. The mean tumor size of Group B (before BOAI) and Group B (after BOAI) was 48.1 mm and 13.6 mm respectively, and Group B consisted mainly of stage IIB patients. The 1998 Annual FIGO Report notes the results of the 5-year survival rate of cervical cancer after surgery, and stage IB1 and stage IIB were 94.5% and 73.0%, respectively. Thus, there was a considerable difference in the 5-year survival rate [26]. However, our results showed no significant difference in 5-year survival rates between Group A and B. This point may indicate the benefits of BOAI followed by NSRH. With regard to prognosis, our study may involve some biases because we excluded some patients who did not take any urodynamic test because they had no difficulty in urinating after 6 months. Fourteen patients who received surgery without BOAI had discontinued the urodynamic test for good results at 6 months after surgery, and 11 patients who underwent surgery after BOAI had discontinued the test for the same reason. Furthermore, forty patients were unrecorded due to various unrelated reasons. Furthermore, some patients in whom BOAI was ineffective were not included in this study. However, in our previous study, we reported the results of prognostic analysis in the population with or without BOAI [22, 23]. The report showed that the NSRH with BOAI group had a longer disease-free survival than the NSRH without BOAI group (*p* = 0.02). However, the overall survival was not significantly different. The relative risk (RR) for recurrence was higher in patients with lymph node metastasis (RR, 4.31; 95% CI, 2.23–8.43) and lower in those who underwent BOAI (RR, 0.30; 95% CI, 0.14–0.68) [23]. These studies suggest the possibility that BOAI does not always affect the prognostic outcome. Since basically most patients with IIB are treated with CCRT, it is hard to compare between the general results. One of the speculated reasons as to why there was no difference between Group A and Group B in terms of the 5-year survival rate might be that both the stage and tumor size of Group B with BOAI became similar to that of Group A and, therefore, we could carry out the complete resection by radical hysterectomy in Group B.

On the other hand, considering the preservation of bladder function, CCRT has advantages compared with surgery. However, Quinn *et al*. reported that the 5-year survival rate of CCRT in stage IIB of cervical cancer was 66% [27]. According to the 1998 Annual FIGO Report, the results of the 5-year survival for surgery and surgery & radiation combined therapy in stage IIB were 73.0% and 64.3%, respectively. Therefore, the 5-year survival rate of CCRT did not yield better results than that of surgery alone [26]. Recent literature has indicated that cisplatin-based concomitant chemo-radiation resulted in superior DFS compared with neoadjuvant chemotherapy followed by radical surgery in locally advanced cervical cancer [28]. However, several patients in this study did not receive complete surgery. 21.5 % of patients crossed over (presurgery crossover and intraoperative unresectable disease) to receive definitive CCRT. In our study, only 6 patients (6.4%) receive presurgical CCRT. Furthermore, all patients who underwent radical surgery received complete surgery. In our institution, the efficiency of BOAI was high, and the 5-year survival rate was also good; therefore, if the performance status of the treatment is favorable, we do not choose CCRT [23]. In addition, since it is impossible for CCRT to be applied to only the cancerous area, there is a possibility that some side adverse effects (bowel dysfunction, fistula, etc.) may continue for a lifetime. Moreover, adjuvant therapy is not always necessary in cases without recurrent risk factors. In this study, adjuvant therapy was not carried out in 27.3% of cases.

Few data have been reported in the literature regarding urodynamic testing after NSRH. Previous studies have shown that, according to urodynamic results, the NSRH technique improves postoperative urinary dysfunction in cervical cancer [29, 30]. Roh *et al*. made a report comparing conventional radical hysterectomy (CRH) and NSRH. The result in this study was that bladder compliance after NSRH was better than that after CRH [30]. Sakuragi *et al*. also reported that the nerve-sparing procedure was successfully completed in 22 of the 27 patients (81.5%). At one year after operation, bladder symptoms had significantly improved in the nerve-sparing group compared to the non–nerve-sparing group [15]. Oda *et al*. reported that the urodynamic study performed at 3 and 6 months after radical hysterectomy showed a statistically significant difference for bladder compliance. Radical hysterectomy with a non-nerve-sparing procedure were risk factors for persistent low bladder compliance (odds ratio [OR], 3.4; 95% CI, 1.1–11.0) [31]. However, patient’s stage in their study was almost limited to IB to IIA. In our study, even for those patients with bulky cervical carcinoma tumors, NSRH became possible by implementing NAC.

On the other hand, urodynamic study is one of the methods to examine the bladder function. There are three types of urodynamic tests: uroflowmetry, cystometrography (CMG), and pressure flow study (PFS). Sakuragi *et al*. and Oda *et al*. used CMG [15, 31]. Although CMG involves the insertion of one catheter into the bladder, PFS involves the insertion of catheters into the bladder and rectum. However uroflowmetry does not require any catheterization and is the least invasive test among the three types of urodynamic tests. Therefore, we chose to use this test in the present study. Uroflowmetry is a test that produces the flow rate of the external urinary stream as volume per unit time in milliliters per second (ml/s). Uroflowmetry reports the maximum flow rate and the volume voided, as well as post-void residual volume. Our study showed that in NSRH after NAC, urine flow was restored equally well as it was in NSRH without NAC (Figure 2). Very few studies have focused on urodynamics after NSRH with NAC. As expected, our study showed that, in early-stage patients who underwent NSRH without NAC, urodynamic study results recovered relatively early. In addition, our results showed that NSRH can be performed on early-stage patients as well, even in bulky tumor cervical cancer cases. Moreover, in those patients who received NAC therapy, tumor volume was also reduced. Postoperative complications and blood loss, however, were not significantly different.

Several limitations of this study should be acknowledged. This study was a Phase II study, not a randomized controlled trial. In addition, some patients abstained from the urodynamic test as mentioned earlier. Patients in whom BOAI was ineffective were not included in this study, as there was no long-term prognosis in such cases.

This study proved that NSRH after BOAI can improve urodynamic results.

In conclusion, according to our analysis, NSRH surgery after balloon occluded arterial infusion NAC brings beneficial results to patients with bulky IB2 to IIB cervical cancers.

## MATERIALS AND METHODS

Patients with cervical cancer who underwent a nerve sparing radical hysterectomy at Osaka Medical College during the period from May 2009 to Nov 2016 entered the study. Patients with locally advanced cervical cancer (stage IB2-IIB) were preoperatively treated with a platinum-based neoadjuvant chemotherapy administered via balloon occluded arterial infusion (BOAI). These patients underwent a urodynamic study preoperatively and at 1 week and 1, 3, and 6 months postoperatively. We excluded patients who did not undergo any urodynamic study at any period or who were found by CT to have metastasis to any lymph nodes before the operation.

### The nerve-sparing radical hysterectomy technique

For the surgery, the abdomen is opened in the standard fashion through a midline incision, the round ligaments are divided, and the broad ligament is opened onto the pelvic sidewall. At our institution, a pelvic lymphadenectomy is usually started before a radical hysterectomy. After the pelvic lymphadenectomy, the ureter is dissected inferiorly, with the encompassing fibro fatty tissue removed and medially reflected to be removed with the uterus. Employing a two point pull-up method [32], we first identify the uterine artery at its origin and use vessel tape to place traction on the artery. Secondly, the uterine artery entering the uterus’ side is identified, and another vessel tape is used to place two points of traction on the uterine artery. Using this two point pull-up method, the connective tissue around the artery becomes unobtrusive, due to the traction, thus allowing us to directly recognize the vessels around the uterine artery. We can also detect the superficial uterine vein and its connection with the bladder - called the superficial vesical vein. We then clamp, ligate and cut each vessel. In this study, we used a bipolar coagulation device and Harmonic1 (Ethicon Endo-surgery Inc, OH, USA). We can also reveal the ureteral branch of the uterine artery which runs from the uterine artery to the ureter cranially, and this vessel is also isolated and ligated. After separating the uterine artery by recognizing it directly, and after ligating the peripheral uterine artery vessels, we carefully divide the anterior leaf of the vesico-uterine ligament. Thereafter, the uterine artery is ligated at its origin. After dividing the anterior leaf of the vesico-uterine ligament, we always divide the uterosacral ligament to make the perirectal space shallower. This process is the key to managing the posterior dissection of the uterus. The same procedure is also performed on the opposite side. Subsequently, we remove the cardinal lymph nodes and divide the cardinal ligaments at the pelvic side wall. When we perform a posterior parametrial resection, the hypogastric nerve and inferior hypogastric plexus are pushed laterally away from the uterosacral and rectovaginal ligaments. After the final resection of the deep layer of the vesico-uterine ligament, we remove the upper third of the vagina.

### Neoadjuvant chemotherapy

Neoadjuvant chemotherapy is administered via balloon occluded arterial infusion (BOAI).

For intra-arterial infusion therapy, we have developed an original 4L-DB catheter (Clinical Supply, Tokyo, Japan) for the simple and efficient injection of an anticancer agent at a high concentration to target spots in patients with advanced uterine cervical cancer. Previously we reported on patients who, under local anesthesia and according to Seldinger’s technique, received polyethylene catheters of 6-French diameter which were inserted through both femoral arteries [22]. Each catheter tip was placed in the internal iliac artery. While the guidewire was detained in the peripheral artery of the internal iliac artery, the catheter was passed through the junction of the uterine artery, which was the target vessel, just distal to the branching out of the superior gluteal artery. To confirm the correct position of the catheter and effective perfusion, a pelvic arteriography was performed during the catheterization procedures. Each time after the completion of treatment, the catheters were removed, and sandbags were used to apply firm pressure over each groin area for 6 h. The regimen included the following: cisplatin 70 mg/m^2^ on day 2 by BOAI, Irinotecan 75 mg/m^2^ on day 1 and 8 by intravenous injection, for two courses every 21 days. Cisplatin was administered intra-arterially within 30 min in divided doses via the bilateral internal iliac arteries. Hydration with normal saline and 5% dextrose began 3 h before chemotherapy, with careful monitoring of urine volume.

### Urodynamic study

The urodynamics in this study considered cystometrography, pressure flow study, and uroflowmetry (UFM). We decided to use UFM because the burden on the patient is mild. Urodynamic studies were conducted before the operation and at 1 week and 1, 3, and 6 months after the operation, and they were carried out using the Flowsky^®^ uroflowmetry device (TOTO) according to the manufacturer’s instructions. One month after surgery, if the residual urine volume was over 100 ml, medication for urinary dysfunction was started. The drugs administered were distigmine bromide (Ubretid^®^) and urapidil (Ebrantil^®^). Dosing was discontinued when the residual urine volume fell below 100 ml.

### Adjuvant therapy

Adjuvant therapy was added when postoperative pathological examinations reveal risk factors for recurrence, including lymph node metastasis, parametrial invasion, deep stromal invasion, lymph vascular space involvement, and bulky tumor (tumor diameter >4 cm). Those patients with high risk factors received concurrent chemoradiotherapy or chemotherapy (paclitaxel and carboplatin).

### Statistical analysis

The statistical analyses were performed using the JMP^®^12 software program (SAS Institute Inc., Cary, NC, USA). The Χ^2^ test and Fisher’s exact probability test were used to evaluate correlations between the urodynamic data and the clinical data. The analyzed clinical outcomes included Qmax (maximum urinary flow rate) and Qave (average urinary flow rate). Dunnett’s test was also used to test for differences between the control group (before 1 week) mean and each exposure group (after 1 month, after 3 months, after 6 months) mean. The analyzed clinical outcomes included overall survival (OS), and overall survival was defined as the time from the first day of treatment to death from any cause. Differences with a *P*-value of less than 0.05 were considered to be statistically significant.

## Abbreviations

NSRH: Nerve sparing radical hysterectomy
NAC: neoadjuvant chemotherapy
UFM: uroflowmetry
BOAI: balloon occluded arterial infusion
CCRT: concurrent chemoradiotherapy
CRH: conventional radical hysterectomy
OS: overall survival
